# Hydroxyapatite-chitosan composites derived from sea cucumbers and shrimp shells ameliorate femoral bone defects in an albino rat model

**DOI:** 10.14202/vetworld.2023.1084-1091

**Published:** 2023-05-24

**Authors:** Arifia Safira, Cinta Atsa Mahesa Rani, Faisal Fikri, Agus Purnomo, Shafia Khairani, Shekhar Chhetri, Salipudin Tasil Maslamama, Muhammad Thohawi Elziyad Purnama

**Affiliations:** 1 Department of Veterinary Science, School of Health and Life Sciences, Universitas Airlangga, Surabaya, Indonesia; 2 Department of Veterinary Surgery and Radiology, Faculty of Veterinary Medicine, Universitas Gadjah Mada, Yogyakarta, Indonesia; 3 Department of Biomedical Science, Faculty of Medicine, Universitas Padjajaran, Bandung, Indonesia; 4 Department of Animal Science, College of Natural Resources, Royal University of Bhutan, Lobesa, Punakha, Bhutan; 5 Department of Agricultural Biotechnology, Faculty of Agriculture, Eskişehir Osmangazi Üniversitesi, Eskişehir, Turkey; 6 Department of Biology, Graduate School of Natural and Applied Sciences, Eskişehir Osmangazi Üniversitesi, Eskişehir, Turkey

**Keywords:** bone defect, chitosan, human and health, hydroxyapatite, sea cucumber, shrimp shell

## Abstract

**Background and Aim:**

A bone defect is defined as a critically sized autologous bone and a bone gap. Bone grafting is one of the most commonly used surgical methods to enhance bone regeneration in orthopedic procedures. A composite of collagen, hydroxyapatite (HA), and chitosan (Ch) is suitable as a bone matrix and stimulates ossification. This study aimed to evaluate the use of natural HA-Ch composites derived from sea cucumbers and shrimp shells and quantify the levels of cytokines, polymorphonuclear neutrophils (PMNs), serum liver enzymes, calcium, phosphate, and procollagen type 1 N-terminal propeptide (PINP) in albino rats with femoral bone defects.

**Materials and Methods:**

A total of 48 albino rats with femoral bone defects were divided into 4 groups (n = 12 each): (C−) placebo, (C+) polyethylene glycol, (T1) HA, and (T2) HA–Ch groups. Each group was divided into two subgroups (n = 6 each), with euthanization on 7- and 42-day post-treatment, respectively. Procollagen Type 1 N-terminal propeptide and the cytokines interleukin (IL)-4, IL-6, IL-10, and tumor necrosis factor-alpha were quantified using enzyme-linked immunosorbent assay. Flow cytometry was performed to evaluate PMNs. A clinical chemistry analyzer was used to measure the serum levels of liver enzymes, calcium, and phosphate.

**Results:**

There was a significant decrease in the level of IL-6 on 7 days and in the level of IL-10 on 42 days in the HA-Ch group. The level of PMNs also decreased significantly on 7 and 42 days in the HA-Ch group. Regarding serum liver enzymes, alkaline phosphatase (ALP) levels in the HA-Ch group increased significantly on 42 days. Calcium and phosphate levels increased significantly on 7 and 42 days in the HA and HA-Ch groups, and PINP levels increased significantly on 7 and 42 days in the HA-Ch group.

**Conclusion:**

The HA-Ch composite derived from sea cucumbers and shrimp shells ameliorated femoral bone defects in albino rats. The HA-Ch composite modulated the levels of IL-6, IL-10, PMNs, ALP, calcium, phosphate, and PINP on 7- and 42-day post-treatment.

## Introduction

In orthopedic terms, bone trauma frequently occurs in the form of fractures, osteoporosis, osteomyelitis, osteosarcoma, and bone defects. Bone trauma cases that affect the femur (28.2%), pelvis (24.8%), mandible (11.4%), tibia (14.8%), radius, and ulna (17.3%) also occur in small animals [1]. Bone defects are discontinuities in the bone and cartilage tissue that are typically caused by injuries and poor healing. Bone defects are commonly observed in several medical cases, including high-grade open fractures with bone loss, severe trauma, blast injuries, complications requiring bone debridement, and resection of bone tumors [2]. In some cases, bone defects cause muscle lacerations, significant bleeding, bone periosteum regression, and soft-tissue damage. Critical bone injuries and multiple fragments are more serious bone regeneration inhibitors than transverse and linear fractures. In addition, delayed union and post-operative complications from bone ossification failure can extend bone defects [3].

Generally, bone defects are easily treated by fixing the bone fragments with intramedullary pins or external fixators. However, small bone fragments are often encountered and difficult to handle; in these cases, a special bone grafting method is required to stimulate the healing process and fill in with a bone substance. Artificial bone grafts or bone substitutes were designed to overcome the limitations of autologous and allogenic bone grafts. Bone substitutes are created using natural bone in an attempt to replicate the mineral profile of bone tissue or the arrangement of interconnected struts in the trabecular bone [4]. Various studies have been conducted on bone treatment methods, including allografts [5], bone grafts [6], and vascularized and non-vascularized bone grafts [7]. However, immunological responses, comorbidity of the donor and recipient status, allograft availability, and potential for disease transmission are crucial factors that must be considered while treating bone defects [8]. On the other hand, developing synthetic materials based on polymers, metals, and ceramics has led to a debate about their efficacy in regenerating, repairing, and replacing bone *in vivo*. Thus, many studies have been conducted during the last decade to formulate biomaterials suitable for accelerating bone regeneration [9].

Xenografts involve using biomaterials to synthesize suitable bone graft materials but have less osteogenic ability than autografts and allografts [10]. Sea cucumbers (*Stichopus variegatus*) and shrimp shells reportedly exhibit the properties of hydroxyapatite (HA), collagen, and chitosan (Ch), which are suitable for cell ingrowth and osteoinduction, have minimal foreign body reactions, and have applications in bone tissue engineering [11, 12]. Hydroxyapatite and collagen fibers are the main components of natural bone. An ideal bone graft material must be able to maintain cell viability, not provoke a high immunological response, increase bone cohesivity, be easy to synthesize, and be noninvasive [13]. Because of the chemical composition and molecular arrangement that are appropriate for the bone matrix and enhanced osteoblast differentiation during osteogenesis, the combination of collagen and HA remains the preferred option [14]. In addition, Ch from shrimp shells exhibits antimicrobial properties and chemotactic activity and is used to increase cell proliferation; moreover, it provides a biodegradable porous structure [15].

This study aimed to evaluate natural HA-Ch composites derived from sea cucumbers and shrimp shells in albino rats with femoral bone defects. To evaluate the efficacy of these biomaterials, we determined the levels of the cytokines interleukin (IL)-4, IL-6, IL-10, and tumor necrosis factor-alpha (TNF-α); polymorphonuclear neutrophils (PMNs); alkaline phosphatase (ALP); aspartate aminotransferase (AST); alanine aminotransferase (ALT); gamma-glutamyl transferase (GGT); calcium and phosphate levels; and procollagen type 1 N-terminal propeptide (PINP).

## Materials and Methods

### Ethical approval

The study was approved by Ethical Committee of Animal Care, Universitas Airlangga with reference No.011/HRECC.FODM/I/2021. Experimental animals were healthy male sexed albino rats purchased from Faculty of Veterinary Medicine, Universitas Airlangga breeding farm.

### Study period and location

This study was conducted for 4 months (March–June 2021). The albino rats were reared at the Animal Laboratory, Faculty of Veterinary Medicine, Universitas Airlangga. Preparation of HA and Ch performed at laboratory animal nutrition. Cytokines quantification, PMN, and PINP levels were observed at the Institute of Tropical Diseases, Universitas Airlangga. Meanwhile, serum liver enzyme, bone calcium, and phosphate levels were performed at Faculty of Veterinary Medicine, Universitas Airlangga.

### Preparation of experimental materials

We collected sea cucumbers and shrimp shells, removed the soft tissues, and cleaned the shells in boiling water. The shells were next dried in an oven at 80°C for 24 h and then subjected to calcination at 200°C for 1 h at a heating rate of 5°C/min to synthesize biogenic HA and Ch powder. The HA gel was prepared by mixing HA with polyethylene glycol (PEG 200 Cat. P3015, Sigma Aldrich, Vienna, Austria) at a ratio of 1:0.25. The HA-Ch gel was prepared by mixing HA with PEG and Ch at a ratio of 1:0.25:0.01 [16]. We also used an amino acid autoanalyzer (L-8900, Hitachi, Japan) to analyze the amino acid composition of HA and HA-Ch (Table-1) [17].

**Table-1:** Amino acids determination in 1 g of HA and HA-Ch.

Amino acid (nmol/cm)	HA	HA-Ch
Aspartic	46.9	29.8
Glutamic	81.2	56.9
Serine	27.6	28.8
Threonine	30.8	19.3
Arginine	48.8	44.8
Glycine	30.9	30.9
Alanine	11.1	11.4
Proline	10.4	12.1
Tyrosine	5.0	2.4
Valine	21.1	16.7
Methionine	5.9	9.5
Cysteine	0.5	0.6
Isoleucine	7.0	8.4
Leucine	16.8	18.9
Phenylalanine	10.3	14.7
Lysine	8.0	41.6

HA=Hydroxyapatite, HA-Ch=Hydroxyapatite-chitosan

### Animal models and surgical procedure

A total of 48 albino rats were acclimatized in individual cages for 7 days and reared under controlled conditions at 27°C with free access to water and a commercial diet. The rats were randomly distributed into four groups (n = 12 each): (C−) placebo, (C+) PEG, (T1) HA, and (T2) HA-Ch groups. Each group was divided into two subgroups (n = 6) and euthanized on 7- and 42-day post-treatment.

Before performing surgery to expose bone defects, all animals were fasted for 12 h and then anesthetized by administering an intramuscular injection of 5 mg/kg body weight (BW) of ketamine, atropine, and xylazine (Ket-A-Xyl® 20 mL, AgroVet, Lima, Peru). A standardized 2-mm diameter and 2-mm deep noncritical bone defect on the lateral-proximal site of the right femur was created using a motorized drill (801G-018, Meisinger, Germany) under saline solution irrigation. The created defects were treated in the assigned groups, sutured with 4–0 nylon monofilament (SN.644, Monosof®, UK), and topically treated with gentamicin sulfate (2–4 mg/kg BW/24 h) [18]. Animal health and behavior were monitored and assessed daily.

### Cytokine quantification

An enzyme-linked immunosorbent assay (R&D Systems, USA) was used to quantify the amount of each cytokine (IL-4, IL-6, IL-10, and TNF-α). A coating buffer mixed with 50–75 μL of capture antibody was filled into high-binding 96-well plates (Greiner Bio-one, Austria) and then incubated overnight at 4°C. An appropriate blocking solution (1% w/v bovine serum albumin) was used to remove the captured antibody and block non-specific binding sites for 1 h at 27°C. The plates were rinsed with phosphate-buffered saline-Tween solution, dried, and then the wells were filled with the supernatant samples in triplicate. The cytokine standard was diluted serially and added to the wells in triplicate. To compare with the blank wells, an assay diluent was loaded into the wells of the respective empty plates and then incubated overnight at 4°C. Biotinylated detection antibody was added to the respective wells, incubated for 2 h at 27°C, and rinsed using horseradish peroxidase-conjugated to streptavidin for 30 min in the dark. The plates were rinsed, and substrate and 1 M H_2_SO_4_ were added to protect the enzyme-mediated color reaction. A microtiter plate reader (VersaMax™, USA) was used to measure the optical density of the color reaction at 450 nm. SoftMax® Pro Software for Windows 10 (SoftMax®, Molecular Devices, California, USA) was used to quantify cytokine levels relative to the blank standard wells [19].

### Evaluation of serum liver enzyme, calcium, and phosphate levels

Serum liver enzymes, namely ALP, AST, ALT, and GGT were analyzed. The remaining serum was collected and stored for 10–20 min in an icebox. We used an automatic clinical chemistry analyzer (Hitachi 902®, Roche Diagnostics, USA) to determine the levels of serum liver enzymes [20].

A standard colorimetric method (Hitachi 902®, Roche Diagnostics) was also performed to determine the serum phosphate and calcium levels. The intra-assay coefficients of variation were 2.5% for serum calcium and 5.6% for serum phosphate [21].

### Determination of PMNs

To determine the PMN percentage, flow cytometry was performed. Polymorphonuclear neutrophils were identified based on side-angle light scattering and forward-angle light scattering, excluding dead cells, other cell-type contamination (such as lymphocytes and erythrocytes), aggregates, and debris from the main analysis. Analysis was performed on Coulter Epics XL (Beckman Coulter®, Villepinte, France) equipped with an argon laser at 488 nm emission and connected to System II software (Beckman Coulter) [22].

### Determination of PINP

Enzyme-linked immunosorbent assay was used to measure the serum levels of PINP (Cloud-Clone Corp., Katy, USA) according to the manufacturer’s instructions. A microtiter plate reader (VersaMax™) was used to determine the optical density of the color reaction, which was compared with the concentration of PINP in the blank sample [23].

### Statistical analysis

All data were expressed as the mean ± standard error and analyzed by performing a one-way analysis of variance followed by the *post hoc* Tukey multiple comparisons test. Values were considered significantly different at p < 0.05. Statistical Package for the Social Sciences v.25 software (IBM, USA) was used for statistical analysis.

## Results

### Evaluation of IL-4, IL-6, IL-10, TNF-α, and PMN

**Figure-1:**
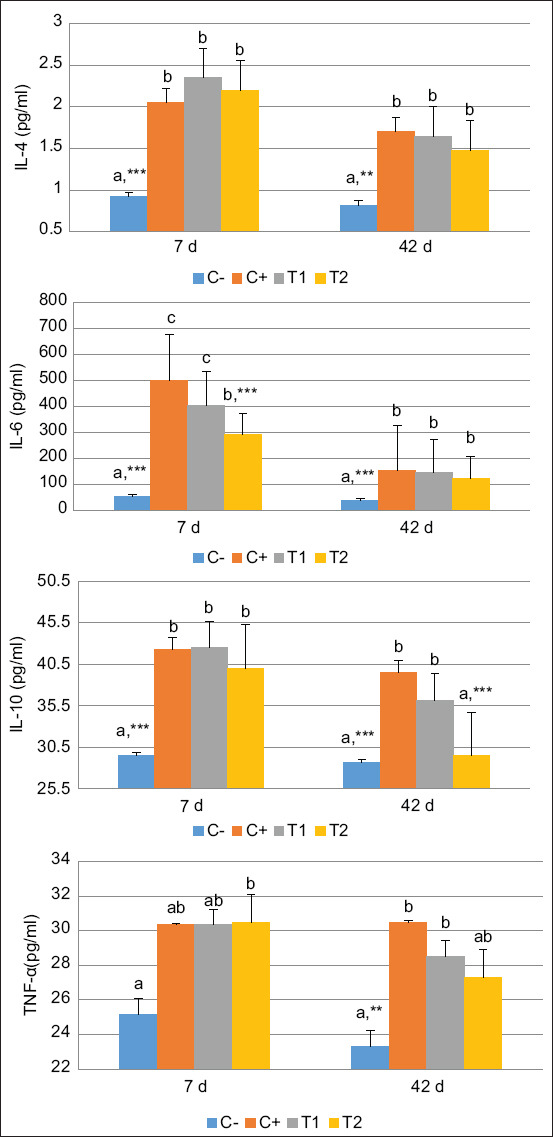
Cytokines evaluation of interleukin (IL)-4, IL-6, IL-10, and tumor necrosis factor-α on 7- and 42-day post-treatment. Values are expressed in mean ± standard error (SE) (n = 6 animals in respective four groups). Values are represented statistically when ^a,b,c^, in comparison with C-group; *p < 0.05, **p < 0.01, ***p < 0.001, in comparison with C+ group.

The levels of all cytokines (IL-4, IL-6, IL-10, and TNF-α) in the PEG, HA, and HA-Ch groups had increased on 7 day, followed by a gradual decrease on 42 day post-treatment. However, the HA-Ch group showed a marked decrease in IL-6 on 7 day and IL-10 on 42 day post-treatment (Figure-1). These findings indicated that cytokine activity modulated inflammation in the initial period of trauma, followed by suppression of IL-6 on 7 days and IL-10 on 42 days after the bone formation period ended.

**Figure-2:**
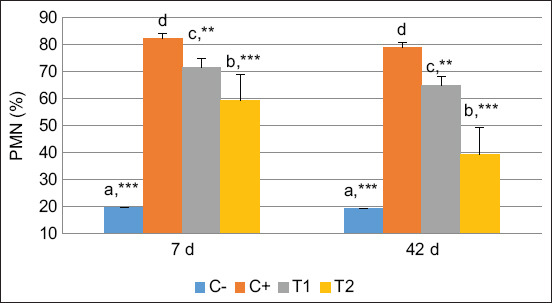
polymorphonuclear neutrophils percentage on 7- and 42-day post-treatment. Values are expressed in mean ± Standard error (n = 6 animals in respective four groups). Values are represented statistically when ^a,b,c,d^, in comparison with C-group; *p < 0.05, **p < 0.01, ***p < 0.001, in comparison with C+ group.

The level of PMNs also showed an increase in the PEG, HA, and HA-Ch groups on 7 days, followed by a decrease on 42-day post-treatment and were significantly lower in the HA group than in the PEG group on 7- and 42-day post-treatment. Moreover, we noted that the level of PMNs was significantly lower in the HA-Ch group than in the HA and PEG groups on 7- and 42-day post-treatment (Figure-2). These results revealed that PMNs and cytokines were detected during the inflammatory phase on 7 days, were prolonged, and then decreased gradually by 42-day post-treatment.

### Evaluation of ALP, AST, ALT, GGT, calcium, phosphate, and PINP levels

**Figure-3:**
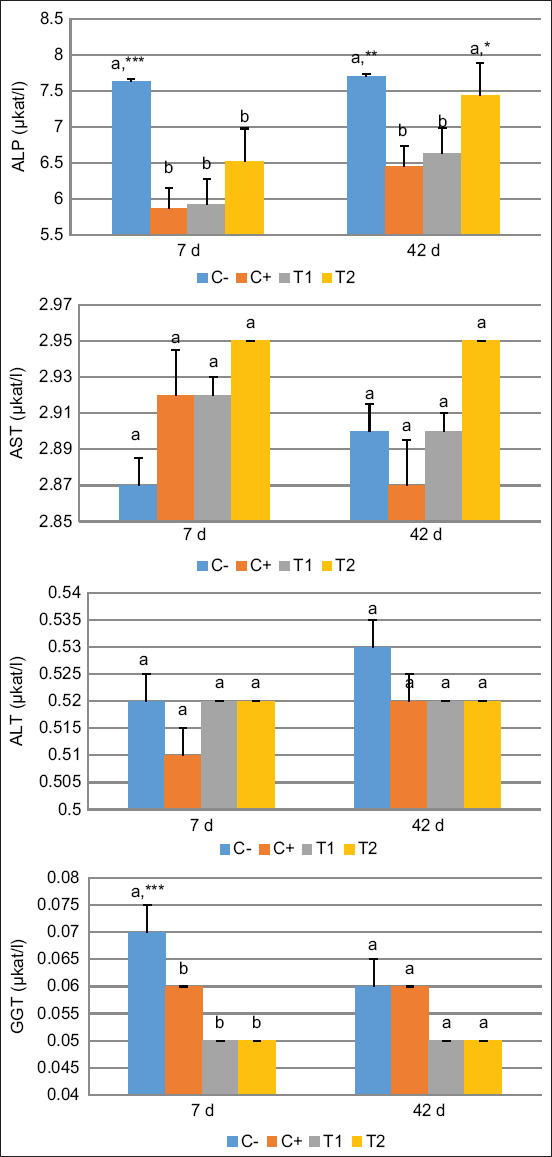
Enzyme serum evaluation of alkaline phosphatase, aspartate aminotransferase, alanine aminotransferase, and gamma-glutamyl transferase on 7- and 42-day post-treatment. Values are expressed in mean ± SE (n = 6 animals in respective four groups). Values are represented statistically when ^a,b^, in comparison with C-group; *p < 0.05, **p < 0.01, ***p < 0.001, in comparison with C+ group.

Serum liver enzyme evaluation showed that only the ALP levels were significantly higher in the HA-Ch group than in the PEG and HA groups on 42 days. No significant differences existed in the AST, ALT, and GGT levels (Figure-3). These findings indicate that ALP was the only major serum liver enzyme present during the bone regeneration period.

**Figure-4:**
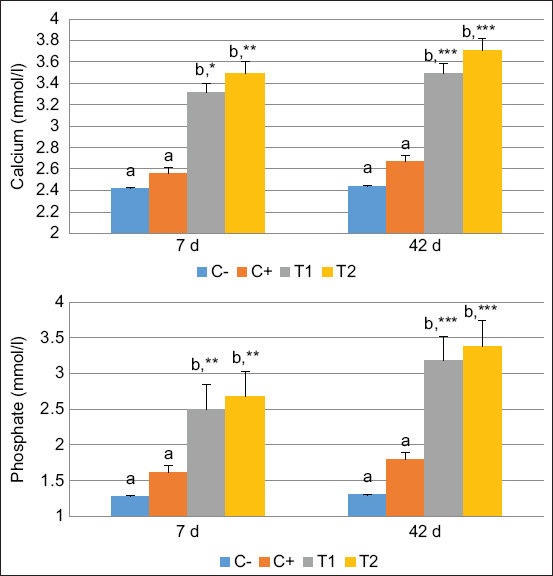
Evaluation of calcium and phosphate levels on 7- and 42-day post-treatment. Values are expressed in mean ± Standard error (n = 6 animals in respective four groups). Values are represented statistically when ^a,b^, in comparison with C-group; *p < 0.05, **p < 0.01, ***p < 0.001, in comparison with C+ group.

**Figure-5:**
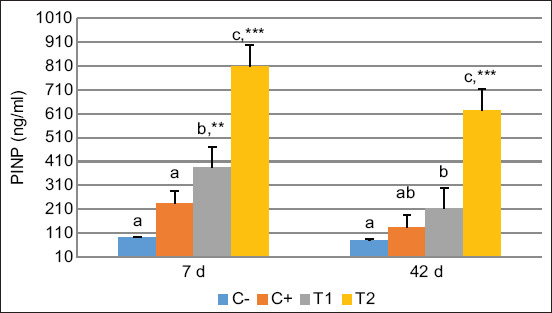
Propeptide of type I collagen level on 7- and 42-day post-treatment. Values are expressed in mean ± Standard error (n = 6 animals in respective four groups). Values are represented statistically when ^a,b,c^, in comparison with C-group; *p < 0.05, **p < 0.01, ***p < 0.001, in comparison with C+ group.

The calcium and phosphate levels were significantly higher in the HA and HA-Ch groups than in the PEG group on 7 days and were constant on 42-day post-treatment (Figure-4). These results indicate that calcium and phosphate play a role in bone regeneration in both the early and termination phases. Calcium and phosphate are natural composites produced during the calcification and mineralization processes. We also determined PINP levels as a biomarker of collagen synthesis in the bone matrix material. The PINP level was significantly higher in the HA-Ch group than in the HA and PEG groups on 7- and 42-day post-treatment, respectively (Figure-5). Procollagen Type 1 N-terminal propeptide activity was significantly associated with ALP, calcium, and phosphate activity during bone regeneration.

## Discussion

Bone grafting is used to restore missing or damaged bone tissue. Bone grafts are primarily used to treat deformed bone that has undergone insufficient post-operative recovery by promoting callus development. Furthermore, the biomaterials used as bone grafts are expected to eliminate the gap in bone continuity by filling in the defective part of the cortex [24]. This study used a natural HA-Ch composite derived from sea cucumbers and shrimp shells as a biomaterial. Hydroxyapatite [Ca_10_(PO_4_)_6_(OH)_2_] has been studied as a potential replacement material for dense connective tissue due to its high biocompatibility and osteoinductivity and has been used clinically as artificial bone and dental implant [25]. However, the clinical value of HA as a bone substitute is a matter of debate; for example, it is difficult to prevent the dispersion of HA granules and mold the granules into a shape that fits the point of the defect [26].

Several previous studies added water-soluble polymers, such as polylactic acid, polyglycolic acid, and sodium alginate, to HA to increase its efficacy [27]. Furthermore, it is claimed that using Ch as a coating material during bone regeneration will improve osteoinduction and cell viability as well as activate and modulate inflammatory cells [28]. Osteoinduction in a scaffold bone graft acts as an adhesive medium for osteoblasts that develop to repair bone defects. In the process of generating woven bones, scaffolding also initiates angiogenesis. The growth within bone apposition from the epiphyseal point to the diaphyseal region can be accelerated by an allograft. Osteoinduction stimulates osteogenesis, thereby implying that bone grafts actively induce stem cells and osteoblasts from surrounding tissues to proliferate and differentiate in the formation of woven bone [29].

Several growth factors are involved in osteoblast differentiation and proliferation, including bone morphogenic proteins, platelet-derived growth factors, insulin-like growth factors, fibroblast growth factors, transforming growth factor-β, and epidermal growth factors [30]. In addition, pro-inflammatory cytokine activity influences the healing process. Cell affinity and viability after biomaterial implantation can be increased if inflammation can be controlled and suppressed. The cytokines involved in the healing process are IL-1, IL-4, IL-6, IL-10, TNF-α, matrix metalloproteinase-9, and prostaglandin E2 [31]. In the present study, IL-4, IL-6, IL-10, and TNF-α increased during early bone defect repair growth. Furthermore, in the HA-Ch group, the activities of IL-6 on 7 days and IL-10 on 42 days decreased during the recovery period. Polymorphonuclear neutrophil levels also increased in the initial period and then decreased gradually by 42 days of the healing period, particularly in the HA-Ch group. A previous study reported that IL-1, IL-6, IL-10, TNF-α, and TGF-β levels indicated an acceleration of the ossification process [32], consistent with the present study’s findings. Another study found that PMNs inhibited osteitis, infection, and possible sepsis surgery by HA-composite implants in patients with femur fractures [33].

In all cases of bone trauma, hemorrhage occurs in the periosteum, endosteum, and soft tissue around the bone. A hematoma forms shortly after the primary trauma, with the release of platelets and macrophages to activate cytokines and promote recovery [34]. On the other hand, reactive oxygen species or free radicals in osteoblasts are inhibited by additional composite polymers in biomaterials [35], which in this study were provided by Ch. The immunosuppressive effect is also supported by the inhibition of NF-κB translocation to the nucleus, which is followed by a decrease in the levels of pro-inflammatory cytokines. Reduced inflammation, enhanced angiogenesis, fibroblasts, and osteoblasts are all supported by the production of pro-inflammatory cytokines during bone defect healing [36]. However, decreased cytokine activity at the end of the healing period indicates well-oxygenated bone trabeculae, optimal osteoblast proliferation, and increased osteocytes and bone matrix compaction [37].

In this study, ALP levels increased on 42-day post-treatment in the HA-Ch group. In addition, the calcium and phosphate levels were significantly higher in the HA and HA-Ch groups than in the PEG group on 7 days and were constant on 42 days. The ALP enzyme plays a greater role than AST, ALT, and GGT, because, during the mineralization phase, ALP provides osteoid tissue, thereby enhancing the adhesion of the outer osteoblast membrane and increasing calcium resorption in bone trabeculae [38]. Alkaline phosphatase is a crucial factor in ossification because it can raise calcium and phosphate concentrations, forming calcium-phosphate bonds with HA that promote bone matrix calcification [39]. In addition to ALP produced by the liver and bones, low levels of ALP can be produced by the intestines, spleen, kidneys, and placenta, indicating disorder in the corresponding organs. Reduced ALP secretion during the healing period of bone trauma indicates termination of the mineralization and calcification phase [40].

During the bone formation phase, PINP concentrations also contributed as a biomarker. There was an increase in PINP concentrations in the HA and HA-Ch groups on 7 days, which decreased gradually on 42-day post-treatment. Clinical testing has shown that during the fracture healing process, some biomarkers of bone regeneration may increase for several weeks after trauma. A previous study [41] also reported a correlation between an early increase in ALP and an increase in PINP. Following a bone defect and throughout fracture healing, biochemical markers of bone turnover may increase for a few months [42]. Procollagen Type 1 N-terminal propeptide is a mature Type I collagen produced from Type I pro-collagen, and the presence of PINP indicates anabolic activity in bone; therefore, PINP is a biomarker of bone formation, so it is recommended that PINP levels be measured during the healing process [43].

## Conclusion

Natural HA-Ch composites derived from sea cucumbers and shrimp shells were effective in promoting bone regeneration in albino rats with femoral bone defects. This study showed that the HA-Ch composite modulated the levels of IL-6, IL-10, PMNs, ALP, calcium, phosphate, and PINP. The serum levels of these biological compounds were measured on 7- and 42-day post-treatment, representing the early and termination periods after the bone defects were initiated, respectively.

## Authors’ Contributions

FF and MTEP: Conceptualized and designed the study. AS and CAMR: Collected the sea cucumber and shrimp samples. AP and SK: Performed sample identification and helped in the visualization and validation of tables and figures. MTEP, AS, CAMR, SC, and STM: Prepared the animal models, surgical procedures, and post-surgical observations. AP, MTEP, and FF: Produced the biomaterials and treated the bone defect. CAMR and FF: Quantified the cytokine level. AS and AP: Investigated the PMNs and PINP levels. MTEP, SC, and STM: Investigated the serum liver enzyme, calcium, and phosphate levels. SK: Helped in the data curation and analysis. SC and STM: Drafted the manuscript. FF, MTEP, and STM: Revised and submitted the manuscript. All authors have read, reviewed, and approved the final manuscript.
